# Risk stratification and contributing factors of deep vein thrombosis among patients admitted at Debre Markos comprehensive specialized hospital, Ethiopia in 2024

**DOI:** 10.3389/fmed.2024.1470212

**Published:** 2024-12-16

**Authors:** Haymanot Zeleke Mitiku, Birtukan Assefa Addis, Afework Edmealem, Dejen Tsegaye, Yalemgeta Biyazin, Abebe Abate

**Affiliations:** ^1^Department of Nursing, College of Medicine and Health Sciences, Debre Markos University, Debre Markos, Ethiopia; ^2^Department of Pediatrics and Child Health, College of Medicine and Health Sciences, Debre Markos University, Debre Markos, Ethiopia

**Keywords:** prevalence, stratification, deep vein thrombosis, Wells Clinical Prediction Model, Ethiopia

## Abstract

**Introduction:**

Deep vein thrombosis is a serious condition and a leading cause of morbidity and mortality in hospitalized patients. Studies conducted in various hospitals in Ethiopia have reported that the prevalence rates of deep vein thrombosis range from approximately 5–10% among hospitalized patients. The risk stratification of deep vein thrombosis and the identification of associated risk factors are critical for assessing deep vein thrombosis in hospital settings. Wells scoring provides a valuable framework for assessing individual risk. This study aims to assess the risk stratification of deep vein thrombosis and to identify the risk factors among patients admitted at Debre Markos Comprehensive Specialized Hospital.

**Method:**

A facility-based cross-sectional study was conducted from December 2023 to February 2024. The study included 423 adult patients, and the data were collected using a structured questionnaire and chart review. Ordinal logistic regression analysis was conducted after performing the model test.

**Result:**

The prevalence of deep vein thrombosis was found to be 7.9%, with 44.2% categorized as having no risk, 8.1% as moderate risk, and 47.7% as high risk for deep vein thrombosis stratification. Various risk factors, such as regular alcohol consumption (odd ratio 4.59, *p* = 0.032), a previous history of deep vein thrombosis (odd ratio 28.32, *p* = 0.000), the presence of a central catheter (odd ratio 12.92, *p* = 0.000), a severe lipid profile (odd ratio 3.8, *p* = 0.001), and a longer duration of stay in the ward (odd ratio 1.28, *p* = 0.000), were significantly associated with high risk for deep vein thrombosis stratification.

**Conclusion:**

The prevalence of high-risk deep vein thrombosis stratification was found to be high, and the occurrence of deep vein thrombosis was also high within this risk group. Regular alcohol consumption, a previous history of deep vein thrombosis, the presence of a central catheter, a severe lipid profile, and a longer duration of stay in the ward were statistically significant for high-risk deep vein thrombosis stratification. This study highlights the importance of identifying patients who are at high risk for deep vein thrombosis stratification and addressing the risk factors for deep vein thrombosis stratification.

## Introduction

Deep vein thrombosis (DVT) occurs when blood clot form in deep veins due to venous stasis, vascular injury, and hypercoagulability, as outlined in Virchow’s Triad since 1856 (1). DVT commonly affects the thighs and lower legs. Once developed, it presents with leg swelling, typically on one side, cramping pain, warmth in the affected area, and skin discoloration (2, 3). Before DVT develops, health professionals should assess patients about their risk status. This allows for effective management through risk stratification, potentially preventing the onset of DVT. One of the assessment methods is to use screening tools such as the Wells Clinical Prediction Model, which aids in categorizing patients into different DVT risk levels (4). This screening tool easily categorizes hospitalized patients into low-risk, moderate-risk, and high-risk stratification. High-risk stratification patients are highly vulnerable to DVT. Similarly, a study indicates that out of 298 patients evaluated, 18 (6%) patients were positive for DVT after utilizing the Wells Clinical Prediction tool (4). A study showed that the incidence of DVT in hospitalized patients increased from 0.8% of admissions to 1.3% of admissions (5). Studies in Brazil (6), Mumbai (2), and Cameroon (3) highlighted varying risks among surgical patients, with DVT being the leading cause of morbidity and mortality. Additionally, 10–30% of patients develop DVT within a month, while sudden death occurs in 25% of untreated cases (7) especially due to pulmonary embolism (8). DVT has long-term complications, such as post-phlebitic syndrome and chronic venous insufficiency, which can significantly affect daily life (9, 10).

Various risk factors contribute to DVT development, including age, body mass index, family planning, immobility, medical conditions (11, 12), nursing interventions, prophylaxis (13), and hematological results, such as hemoglobin, D-dimer, cholesterol level, and bilirubin. Nurses play a pivotal role in mitigating DVT risk through interventions, such as using compression stockings, anticoagulant administration, and promoting blood flow through massage (14, 15).

In Ethiopia, specifically at Debre Markos Comprehensive Specialized Hospital, despite the known hazards and problems, there is still a significant gap in the application of preventative interventions, particularly the use of trustworthy screening methods such as the Wells Clinical Prediction Model. This method, which has been shown to be effective in categorizing individuals as low, moderate, or high risk, has the potential to significantly reduce the incidence of DVT through early detection and intervention. However, based on the observations and feedback from clinical practice, nurses, and healthcare professionals at this hospital, this method is yet to be incorporated into routine assessments. The present emphasis on treating patients with pre-existing DVT rather than preventing its emergence exposes many hospitalized patients particularly those in high-risk categories to this life-threatening illness.

Furthermore, the lack of consistent prevention strategies, such as the use of compression stockings, anticoagulants, and other nursing interventions, exacerbates the problem. Studies have shown that without such interventions, the risk of DVT increases, particularly among patients with additional risk factors such as immobility, advanced age, and underlying medical conditions (16–18). By assessing the risk stratification of DVT, the prevalence of DVT, and associated factors among patients admitted at DMCSH, the findings can provide critical insights into the scope of the problem and the necessary interventions required. This research could also serve as a foundation for advocating the implementation of screening tools such as the Wells Clinical Prediction Model, and other evidence-based preventive measures within Ethiopian health institutions, potentially saving lives and reducing the burden of DVT-related complications. Therefore, this study aimed to assess the risk stratification of DVT, the prevalence of DVT, and associated factors among admitted patients at Debre Markos Comprehensive Specialized Hospital in 2024.

### Conceptual framework

**Figure 1:**
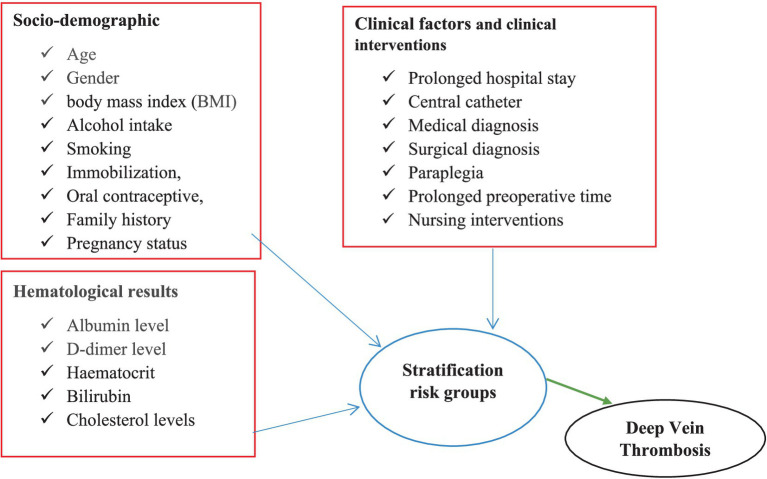
Conceptual framework of DVT risk stratification, prevalence of DVT, and clinical factors among medical and surgical patients admitted at DMCSH.

This framework illustrates the relationship between various independent variables, including socio-demographic factors, clinical characteristics, clinical interventions, and hematological findings, with the dependent variable of DVT risk stratification. This framework was developed after a thorough review of multiple studies and literature sources (Figure 1).

## Materials and methods

### Study area and period

This study was conducted at Debre Markos Comprehensive Specialized Hospital in Debre Markos, the administrative capital of the East Gojjam region, from 28 December 2023 to 1 February 2024. Debre Markos is located 295 km from Ethiopia’s capital, Addis Ababa, and 265 km from Bahir Dar, the capital of the Amhara regional state. The hospital has 213 admission beds, including 44 in the medical ward and 36 in the surgical ward. It is staffed by 196 nurses, 49 physicians, and 211 other professionals. The maximum patient stay at the hospital was 18 days in the medical ward and 30 days in the surgical ward, with an average of 180 and 160 monthly admissions, respectively. The hospital provides 24-h services daily and serves a catchment area of more than 3.5 million people, with over 2,210 annual admissions.

A facility-based, cross-sectional study was conducted to assess the risk stratification of DVT, the prevalence of DVT, and its associated factors among the patients admitted at Debre Markos Comprehensive Specialized Hospital. All adult patients admitted to the medical and surgical wards at the time of data collection were considered the study population. All adult patients admitted for 48 h or more (12) in the medical and surgical wards at Debre Markos Comprehensive Specialized Hospital were included in the study. Patients admitted to the medical and surgical wards due to a diagnosis of DVT were excluded from the study.

The sample size was calculated using a single proportion formula based on the following assumptions: since there was no previous study conducted in Ethiopia, the maximum sample size was taken. The proportion of DVT risk stratification was assumed to be 50%, with a 95% confidence level, a 5% margin of error, and a 10% non-response rate. The final sample size was 423 participants.

### Sampling procedure

All eligible adult patients admitted to the medical and surgical wards at Debre Markos Comprehensive Specialized Hospital were included. The monthly hospital admission report showed 180 patients in the medical ward and 160 patients in the surgical ward, with average daily hospital discharges of 6 and 5 patients, respectively. Two months of data were considered, totaling 360 patients in the medical ward and 320 in the surgical ward. Proportional allocation was applied. Each day, all eligible patients were recorded, and random selections were made until 198 surgical and 224 medical patients were chosen. The k values of 1.88 ~ 2 and 1.5 ~ 2 were selected daily for patients admitted in medical and surgical wards, respectively, through a lottery mechanism after listing their bed numbers. Data collection continued until reaching the desired sample size and the data were collected until patient discharge.

### Operational definition

Stratification of DVT risk was assessed with the Wells’ checklist: 0 signifies no risk, 1–2 signifies moderate risk, and ≥ 3 indicates high risk.

DVT diagnosis was confirmed through Doppler ultrasound for both symptomatic patients and those suspected of DVT, based on the physician’s clinical decision. In cases where Doppler ultrasound was not feasible for various reasons, prothrombin time was used, with a cutoff point of >15 s determined by the physician (19).

Alcohol consumption was confirmed by simply asking the participants their status on alcohol intake. Those who had never consumed alcohol or had completely ceased alcohol intake for the past year were categorized as “never take alcohol.” Those who reported drinking frequently were classified as “regular alcohol consumers,” while those who drank occasionally or only during holidays were categorized as “occasional alcohol consumers.”

Patients who reported never having smoked cigarettes were categorized as “non-smokers,” while those who had smoked cigarettes in the past month were categorized as “smokers.”

Immobilization status was defined as patients being unable to move from the bed and totally dependent on others for performing personal activities such as bathing, eating, positioning, grooming, etc.

### Variables of the study

#### Dependent variable

Stratification of DVT risk (low, moderate, and higher).

#### Independent variables

##### Socio-demographic factors

Age, sex, occupation, residence, BMI, alcohol intake, smoking, immobilization, and pregnancy and postpartum status were under the socio-demographic variable.

##### Clinical factors and hematological laboratory results

Central catheter, medical and surgical diagnoses, paraplegia, major surgeries (hip, femur, and knee surgery), trauma, prolonged stay in the ward, albumin levels, cholesterol levels, and nursing interventions (such as compression stockings, anticoagulant administration, and promoting blood flow through massage or range-of-motion exercises) were the variables included.

#### Data collection procedure and tools

Despite various assessment options available, risk assessment models, such as the Wells Clinical Prediction Rule, are valuable and highly recommended tools for accurately identifying risk stratification of DVT among admitted patients (4). Patients’ charts were reviewed, and interviews were conducted to collect demographic details (such as age, gender, place of residence, lifestyle factors such as smoking, alcohol consumption, exercise level, medical and surgical diagnoses, and BMI).

In this study, laboratory results for participants’ albumin and cholesterol levels were the included. For patients with multiple laboratory tests, the one closest to data collection was used for analysis. The BMI (kg/m^2^) categories were defined as normal, underweight, or overweight according to the WHO criteria. Four general practitioners were recruited as data collectors, with one M.Sc. Nurse serving as supervisor. They underwent a day of training on the study’s purpose, checklist completion, measurements, and ethical considerations to ensure standardized data collection. Supervisors and investigators monitored the data collection process.

### Data quality control

Data quality was maintained through a standardized data extraction tool. A pre-test was conducted on 10% of the sample (42 patients). Data collectors were trained on the tool and data collection procedures. The supervisor closely monitored the data collection by conducting daily evaluations for completeness and promptly addressing any issues. The investigator checked all the collected data for completeness and consistency during management, storage, and analysis. The patients at high risk of DVT underwent ultrasound and laboratory testing on the same day as data collection.

### Data processing and analysis

After data collection, inconsistencies and missing values were addressed. Frequency analyses, summary statistics, and visualizations were performed to detect outliers, duplications, or unexpected patterns, helping to identify data entry errors. For continuous variables, mean/median imputation was applied, while predictive imputation was used for categorical variables. Then, the data were entered into Epi Data Manager software version 4.2. Analysis was performed using SPSS window version 25, employing descriptive and summary statistics to characterize study variables. Ordinal logistic regression identified risk factors for the risk stratification of DVT, with factors having a *p*-value of ≤0.25 in the bivariate regression analysis included in the final model. Statistical significance was set at *p* < 0.05 with a 95% CI. Model fitness was assessed across three assumptions of ordinal logistic regression. The model fitting information showed a *p*-value of 0.000, fulfilling the assumption that the dependent variable is ordinal and can perform an ordinal logistic regression model for this data. The parallel line test yielded a *p*-value of 0.764, which is greater than 0.05, meeting the assumption of proportional odds. Finally, there was no significant multicollinearity; all the variables had a tolerance of less than 10.

## Results

### Descriptive results

In the socio-demographic characteristics of the patients admitted to the medical and surgical wards, a response rate of 405 was achieved out of 423 participants who took part in the study. Among these respondents, 216 were medical patients, while the remaining were surgical patients. Out of the 405 responders, 172 were men, 224 were urban residents, and 158 were married. Approximately 27.9% were aged between 41 and 50, with 48.28 mean ages, 116 were farmers, 67 smoked cigarettes, and 226 occasionally consumed alcohol (Table 1).

**Table 1:** Socio-demographic characteristics of the risk stratification of DVT, and DVT prevalence among patients admitted at Debre Markos Comprehensive Specialized Hospital, (*N* = 405), in 2024.

Variable	Categories	Frequency	Percentage (%)
Sex	Male	172	42.5
	Female	233	57.5
Age	Age 18–30	54	13.3
	Age 31–40	72	17.8
	Age 41–50	113	27.9
	Age 51–60	83	20.5
	Age above 60	83	20.5
Residence	Urban	224	55.3
	Rural	181	44.7
Marital status	Married	158	39.0
	Divorced	62	15.3
	Single	107	26.4
	Widowed	78	19.3
Educational status	Cannot read and write	98	24.2
	Up to Primary school	179	44.2
	Up to Secondary school	78	19.3
	College and above	50	12.3
Occupation	Farmer	116	28.6
	Merchant	96	23.8
	Student	47	11.6
	Daily laborer	71	17.5
	Government employee	75	18.5
Alcohol consumption	Occasionally	226	55.8
	Regularly	142	35.1
	Never takes alcohol	37	9.1
Cigarette smoking	Current Smoker	67	16.5
	Non-smoker	208	51.4
	Ex-smoker	130	32.1
Mobility	Cannot move	120	29.6
	Legs cannot move	136	33.6
	Perform daily activities	149	36.8
Transferred from other hospitals	No	161	39.8
	Yes	244	60.2
Paralysis of lower extremities	Yes	22	5.4
	No	383	94.6
Central catheter	Yes	89	21.0
	No	320	79.0
Change position	Supine for <2 h	116	28.6
	Leg overlap for >2 h	42	10.4
	Change position frequently	247	61.0
Cholesterol level	normal level	157	38.8
	abnormal level	128	31.6
	Unknown	120	29.6
History of DVT	Yes	128	31.6
	No	205	68.4
DVT	Developed DVT	56	13.8
	Not developed DVT	349	86.2
Location of DVT	Proximal DVT	18	32.1
	Distal DVT	38	67.9
Breathing	Yes	228	56.3
	No	177	43.7
Anticoagulant	Yes	225	55.6
	No	180	44.4
Leg elevation	Yes	269	66.4
	No	136	33.6
Exercise	Yes	232	57.3
	No	173	42.7
Massage	Yes	274	67.7
	No	131	32.3
Stratification	No risk	179	44.2
	Moderate risk	33	8.1
	High risk	193	47.7

**Figure 2:**
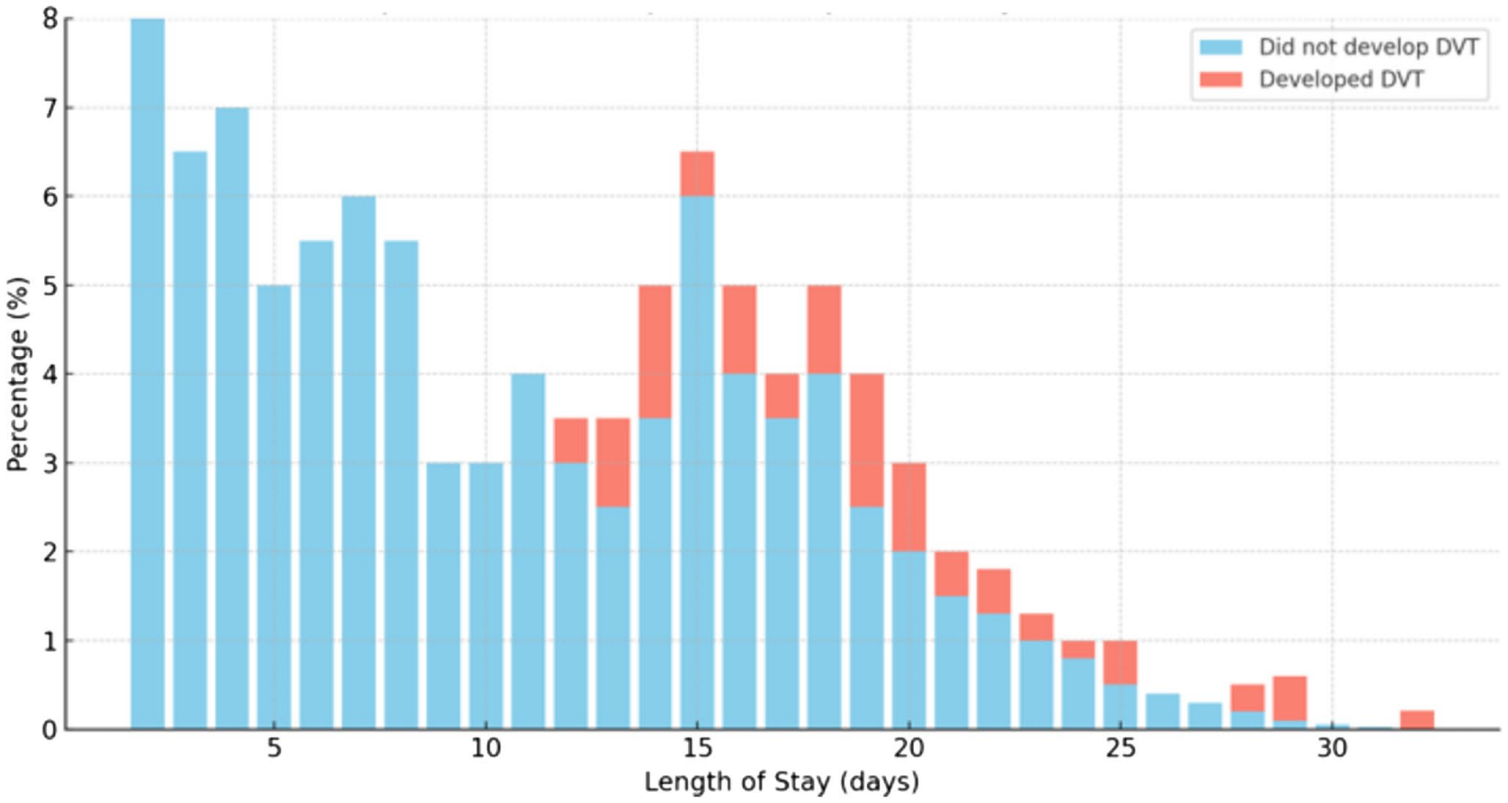
The chance of developing DVT due to the duration of stay in the ward among medical and surgical patients admitted at Debre Markos Comprehensive Specialized Hospital, (*N* = 405), in 2024.

The mean duration of stay in the ward was 12.22 days, with a minimum of 2 days and a maximum of 32 days. Those who had a short duration of stay in the ward had a low chance of developing DVT (Figure 2). Figure 2 shows that no patients developed DVT up to 11 days of hospital stay; however, cases of DVT were observed after this period. In another ward, the patients did not develop DVT during the early days of admission as well.

**Figure 3:**
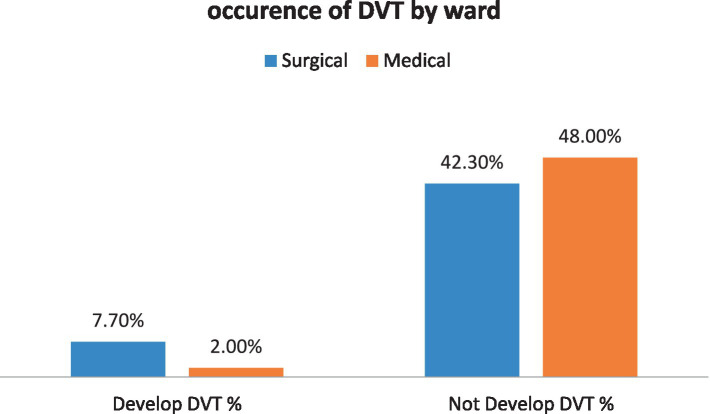
The proportion of patients developing DVT due to ward difference among patients admitted at Debre Markos Comprehensive Specialized Hospital, (*N* = 405), in 2024.

Furthermore, the data from this study demonstrates that participants in surgical wards have a higher percentage of having DVT than those in the medical wards (Figure 3).

**Figure 4:**
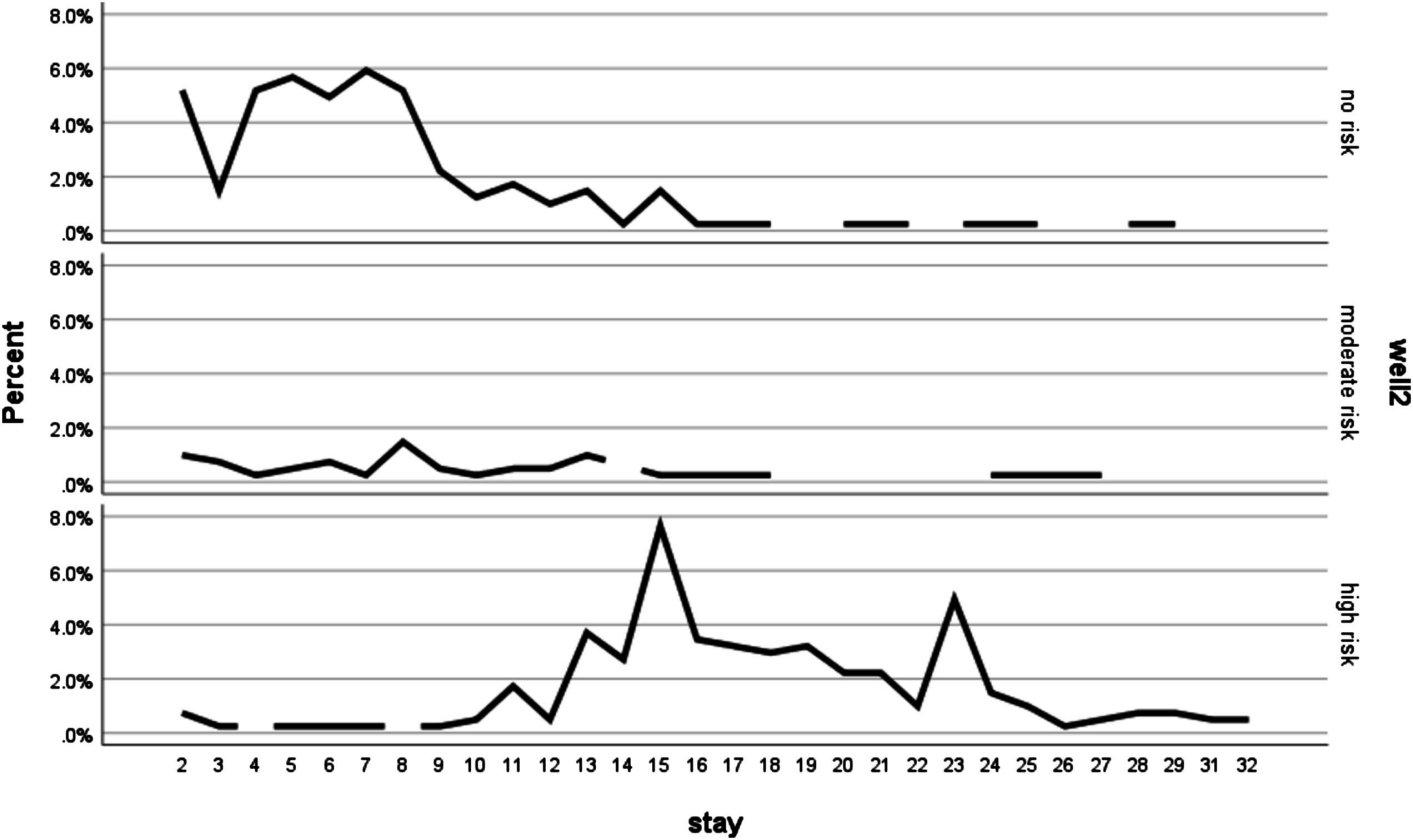
The proportion of DVT stratification due to the duration of stay in the ward among patients admitted at the Debre Markos Comprehensive Specialized Hospital, (*N* = 405), in 2024.

Additionally, this study indicates that individuals who had a long duration of stay in the ward had a high chance of being in the high-risk DVT stratification group. Furthermore, there was a high chance of being in the high-risk DVT stratification group, especially after staying 13 days to 23 days in the ward. Then, after 23 days of staying in the ward, there was a similar chance of being in the all-different risk DVT stratifications (Figure 4).

**Figure 5:**
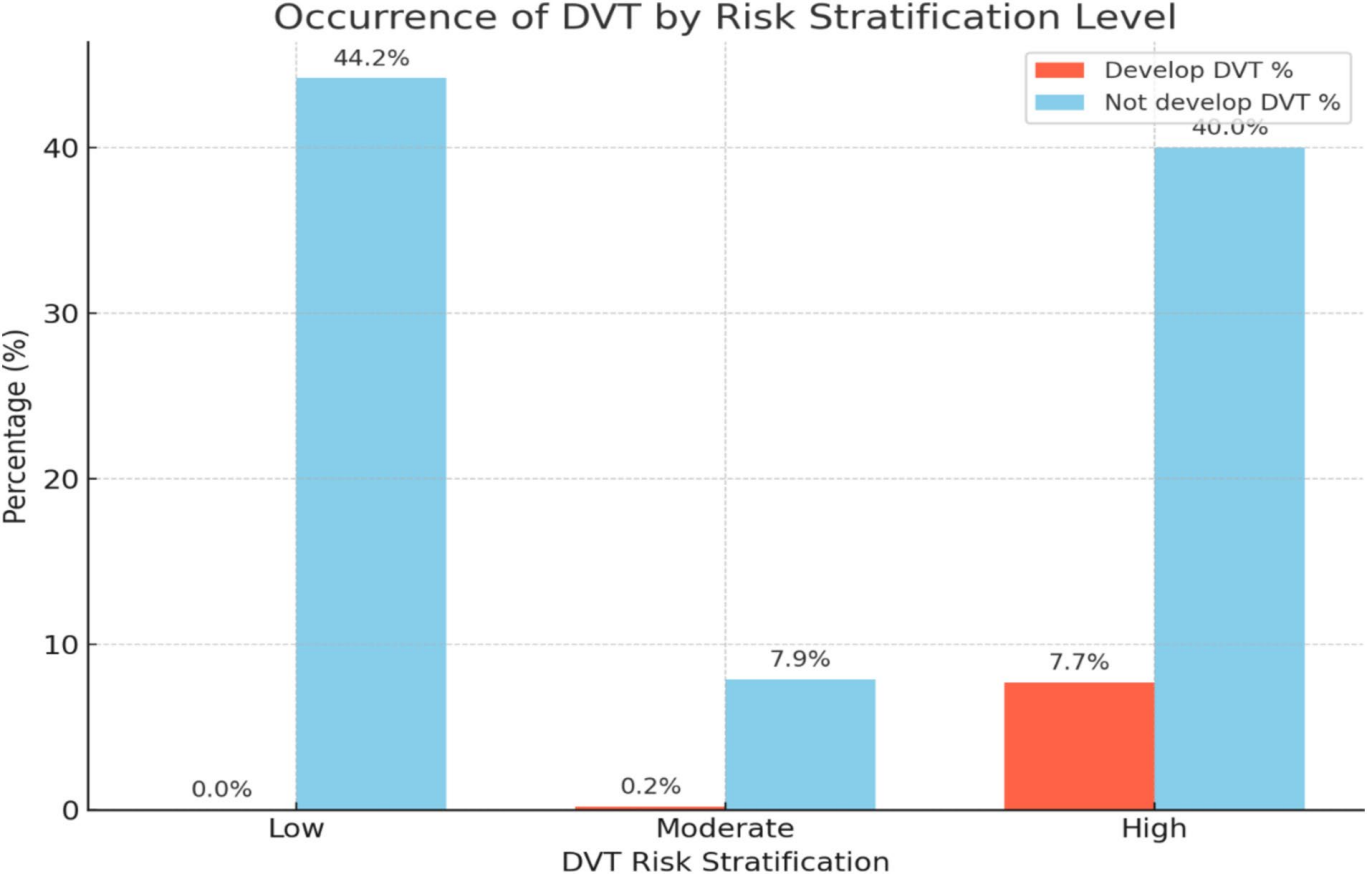
A bar graph illustrating the occurrence of DVT by risk stratification level among patients admitted at Debre Markos Comprehensive Specialized Hospital, (*N* = 405), in 2024.

Overall, the occurrence of DVT was confirmed in 32 cases (7.9%) out of 405 participants through Doppler ultrasound or by the physician’s clinical decision with the guidance of the laboratory investigation. Nearly all the occurrences of DVT were from the high-risk DVT stratification group (Figure 5).

### Factors associated with stratification of DVT risk

Variables with a *p*-value of ≤0.25 in the bivariable analysis were entered into the final model. Then, sex, age, educational status, occupation, alcohol and smoking status, being transferred from another health institution, history of previous DVT, length of stay in the ward, having a central catheter, and cholesterol levels were found to be significant predictors for the risk of DVT. Subsequently, the final ordinal logistic regression model was conducted, and the variables were considered statistically significant when the *p*-value was less than 0.05 with a 95% confidence interval (CI).

Regular alcohol consumers face 4.59 times high-risk DVT stratification compared to non-consumers, with an 82% probability of high-risk DVT stratification. Conversely, the likelihood of not being at high-risk DVT stratification for regular alcohol consumers stands at 18%. Being transferred from another hospital escalates the risk by 43.2 times, with a 97.7% probability of high-risk DVT stratification, leaving only a 2.3% chance of not being at high-risk DVT stratification. Similarly, individuals with a history of previous DVT have 28.32 times high-risk DVT stratification, with a 96.6% probability of being at high-risk DVT stratification, and a 3.4% probability of not being at high-risk DVT stratification. Cholesterol levels above the normal range increased the risk by 3.8 times, with a 79.2% probability of high-risk DVT stratification, and a 20.8% probability of not being at high-risk DVT stratification. Having a central catheter raises the risk by 12.92 times, with a 92.8% probability of high-risk DVT stratification, and a 3% probability of not being at high-risk DVT stratification. By increasing one day of stay in the ward, the likelihood of high-risk DVT stratification was increased by 1.2 times, with a 54.5% probability of being classified as high risk (Table 2).

**Table 2:** The factors associated with the risk stratification of DVT in ordinal regression analysis among medical and surgical patients admitted at Debre Markos Comprehensive Specialized Hospital, (*N* = 405), in 2024.

Variable		Stratification of DVT risk			B	Exp(B)	*p*-value
		Low	Moderate	High			
Sex	Male	86	11	75	−0.57	0.56	0.193
	Female	93	22	118		1	
Educational status	Cannot read and write	49	9	40	−0.371	0.690	0.591
	Up to Primary school	88	15	76	−0.936	0.392	0.163
	Secondary school	33	7	38	0.193	1.213	0.793
	College and above	9	2	39		1	
Occupation	Farmer	75	7	34	0.232	1.261	0.696
	Merchant	58	5	33	−0.003	0.997	0.996
	Students	20	7	20	−0.907	0.404	0.225
	Daily laborers	13	7	51	0.487	1.627	0.451
	Governmental employed	13	7	55		1	
Alcohol consummation	Occasionally	123	12	91	0.429	1.535	0.532
	Regular	38	17	87	1.525	4.596	*0.032*
	Never	18	4	15		1	
Smoker status	Current smoker	29	5	33	0.385	1.470	0.512
	Non-smoker	77	21	110	−0.167	0.846	0.718
	Ex-smoker	73	7	50		1	
Transferred from other hospital	Yes	6	8	147	3.76	43.24	*0.000*
	No	178	25	41		1	
History of previous DVT	Yes	8	6	114	2.911	28.32	*0.000*
	No	179	27	71		1	
Cholesterol	Above normal range	47	12	98	1.346	3.843	*0.001*
	Unknown	68	8	44	2.911	3.76	0.731
	In normal range	70	11	47		1	
Have central catheter	Yes	8	6	75	2.55	12.92	*0.000*
	No	178	27	111		1	
Age category	18–30	26	12	16	0.590	1.026	0.803
	31–40	35	4	33	0.512	1.286	0.782
	41–50	50	6	57	0.355	1.804	0.786
	51–60	38	5	40	0.599	1.668	0.501
	>60	30	6	47		1.426	
Stay in the ward					0.250	1.28	*0.000*
					0.017	1.017	0.217

NB: *significant (*p*-value < 0.05); 1: as reference. The italic values indicates significant factors.

## Discussion

In this study, the occurrence of DVT was 7.9%, with a CI of 5.1–10.7%. This finding aligns with the results of other studies: a retrospective study conducted in Hawassa (20) showed that 10.6% developed DVT out of all admitted patients; in China (21), a 6.4% rate was observed among patients admitted to surgical ward; and in Rwanda (22), a 5.5% incidence was reported among medical and obstetrics–gynecology inpatients. Additionally, an Italian study found a 7.07% DVT rate detected through CT angiography in patients admitted to the ward (23), while a study from Germany (24) reported a 7.8% incidence of DVT among non-surgical intensive care unit patients. However, it is important to note that nearly all of these studies were conducted in different study populations, which may have varying characteristics, such as demographics, underlying health conditions, and clinical settings. Furthermore, the methodological differences, such as the diagnostic tools used (e.g., CT angiography in the Italian study), could impact DVT detection rates. The Italian study’s reliance on CT angiography may have led to more accurate detection compared to studies using lesser sensitive methods. Additionally, clinical settings, such as surgical wards versus non-surgical intensive care units, could contribute to the variability in DVT rates.

This study reports a higher rate compared to a study conducted in India, where the DVT incidence was 4.3% among high-risk Indian neurosurgical patients (25). The difference perhaps was due to the measurement used. In this study, Doppler ultrasound was used, and also for those not feasible for various reasons, the prothrombin time was used, with the clinical decision of the physician. However, the comparative study used images that were more accurate. This might have enabled the outcome.

However, in this study the prevalence is lower than those reported in previous studies. A meta-analysis conducted in India (26) found a prevalence of 12.8% among patients with spinal cord injury. A large, prospective epidemiologic study conducted in France reported a prevalence of 24.9% (27), while Zambia reported 11.1% among medically admitted patients (28). Additionally, in England, focusing on advanced cancer palliative care, the DVT prevalence rates were revealed to be 36% (29), while a meta-analysis showed that 14.8% (26) DVT prevalence occurred among patients with spinal cord injury. These discrepancies occurred because the study targets/populations were different. In this study, patients who had DVT upon admission or had hospital stays of less than 2 days were excluded. Additionally, in this study, the patients admitted for the case of DVT were also excluded. All these could underestimate the prevalence of DVT.

In this study, the stratification of DVT risks was classified into three, they are no risk, moderate risk, and high risk of DVT. The results showed that 44.2% of the hospitalized patients had no risk, 8.1% had moderate risk, and 47.7% had a high risk of DVT. Among those identified as high risk of DVT, 7.7% developed DVT, accounting for 99.8% of all DVT cases in this group. The rest were from the moderate risk group of DVT stratification. For example, a study in the US found that 64% of admitted patients underwent risk stratification, with 36% classified as high-risk and low-risk for DVT, respectively. Among the high-risk group, 10.8% developed DVT (30), which is really similar to this study.

Another study conducted in Indiana, USA, found that the clinical probability score for the stratification of DVT risk was 21% low, 40% moderate, and 39% high (31). The difference decidedly occurred because of the tool they used and the population difference.

Furthermore, a cross-sectional observational study conducted in Brazil showed that DVT risk groups consisted of 8.4% low risk, 17.3% moderate risk, 29.7% high risk, and 44.6% were classified as very high risk (6). Another study conducted at an Israeli hospital showed that DVT risk groups consisted of 73.8% low risk and 26.1% high risk (32). Similarly, a study in Japan revealed that the stratification for DVT risk was as follows: 0% very low risk, 21.9% low risk, 66.7% moderate risk, and 11.4% high risk (33). Another study conducted in Mumbai, India, revealed that the mean risk score for DVT was 5.15, with 67.56% classified as having a high to very high risk for DVT (2). Another study in India revealed that 4.5% were at no risk, 41.8% were at moderate risk, 6% were at high risk, and 23.9% were at the highest risk of developing DVT. Additionally, based on the pre-test probability scores, 76% were classified as low risk, 20.9% as moderate risk, and 3% as high risk (12). The difference is attributed to variations in tools used, study target, and demographic factors.

In this study, the odds of regular alcohol consumers being at a higher risk of developing deep vein thrombosis (DVT) are 4.59 times greater than those who do not consume alcohol. This finding aligns with similar studies conducted in India and Germany, where frequent alcohol intake was also associated with an elevated risk of DVT. These studies suggest that regular alcohol consumption may contribute to changes in blood viscosity, coagulation, or vascular inflammation, all of which are factors that can increase the likelihood of DVT. Consistent with these results, our study underscores the importance of considering alcohol intake as a significant risk factor in DVT prevention and patient risk stratification (24, 26). This is probably due to alcohol consumers having low immune status, thus delaying the recovery period from their diseases. Other variables in this study associated with high-risk DVT stratification included patients transferred from other hospitals and those with longer ward stays. The likelihood of increased DVT risk for patients transferred from other hospitals is 97.7%, while patients with longer ward stays had a 1.2 times higher risk compared to those with shorter stays. These findings are consistent with other studies that have shown a significant association between long-term immobility, extended hospitalization, and DVT (24, 26). This is most likely due to prolonged ward stays that cause changes in blood flow, vascular endothelial injury aggravated by medication, disease processes, or immobility. In an India study, prolonged intra-operative supine and lateral lying positions exceeding 5 hours, severe postoperative motor deficits, and ambulation delays exceeding 2 days were observed as significant DVT risk factors (26). Infectious diseases, prolonged preoperative periods, trauma, malignant neoplasms, organ failure, heart failure, hypertension, respiratory failure, and cancer are associated with an increased risk of DVT (21, 25, 26, 34, 35). These conditions can lead to tissue destruction, which in turn promotes the aggregation of thrombosis (36).

According to this study, having a central catheter raises the risk of DVT by 92.8%, while having a past history of DVT increases the risk by 96.6%. These results are corroborated by a study carried out in the US that also discovered substantial correlations between DVT and patients who had central catheters, a history of DVT, or an underlying thrombophilic disease. If a prior DVT reveals a risk for clot formation, such as vascular injury or persistent prothrombotic tendencies, the chance of recurrence may be elevated. The venous wall may become irritated or damaged by a central catheter, which could lead to the development of localized clots (31, 34, 37).

In this study, the odds of having cholesterol levels above 300 mg/dL were 3.8 times higher for high-risk DVT stratification than those with cholesterol levels within the normal range. The probability of cholesterol above the normal range leading to a higher risk of DVT stratification is 79.2%. This is supported by a study in China, which found that high cholesterol levels are associated with DVT (38). When plasma cholesterol levels are elevated, it can lead to increased activation of coagulation pathways and promote inflammation and fatty streak formation (39).

A study conducted in the US indicates that individuals aged over 75 years have an increased risk of developing DVT (34), but is not associated with this study. Other studies showed that an increase in age by 10 years and being male were associated with DVT (21, 35). However, nursing care, such as the use of compression stockings or other devices, administering prophylaxis, changing lying positions every 2 h, encouraging exercise, and elevating the legs can significantly reduce the risk of DVT (14, 15, 40, 41).

## Conclusion

The prevalence of high-risk DVT stratification was found to be high, and the occurrence of DVT was high within this risk group, necessitating close attention. Factors such as regular alcohol consumption, a history of previous DVT, the presence of a central catheter, severe lipid profile, and a longer duration of stay in the ward were statistically significant in association with high-risk DVT stratification.

### Recommendations

This study on the stratification of DVT risk and DVT among patients admitted at Debre Markos Comprehensive Specialized Hospital suggests several recommendations:

1. Adoption of Wells Clinical Prediction Model: Implementing this model can aid in the early identification of high-risk patients, enabling timely intervention.
2. Enhanced Prevention Strategies: Priority should be given to preventive measures for patients at high risk of DVT stratification, as they are particularly vulnerable to developing DVT. Interventions such as the use of compression stockings, early mobilization, and appropriate anticoagulant therapy may be necessary for these patients.
3. Regular Screening and Monitoring: Continuous assessment and follow-up of those who are at high risk of DVT stratification are crucial for early detection and management.
4. Multidisciplinary Approach: Collaboration among healthcare professionals ensures holistic care plans for high-risk DVT stratification patients. Facilitating patient recovery to minimize patient hospitalization, central catheterizing, and reducing cholesterol levels require a multidisciplinary approach.

### Limitations of this study

The major limitation of this research is the use of outdated citations, which is due to the scarcity of studies conducted and the difficulty in finding more recent studies to cite.

## Data Availability

The raw data supporting the conclusions of this article will be made available by the author, HM, with email address of haymanotzeleke89@gmail.com, without undue reservation.

## References

[1] Wessler S Reimer SM Sheps MC. Biologic assay of a thrombosis-inducing activity in human serum. J Appl Physiol. (1959) 14:943–6. doi: 10.1152/jappl.1959.14.6.943 13844118

[2] Palkar AV Karnik ND. Risk stratification, prevalence by hand-held microdoppler and in-hospital mortality of deep venous thrombosis in indoor geriatric population. J Assoc Physicians India. (2013) 61:539–42. PMID: 24818337

[3] Nkoke C Tchinde Ngueping MJ Atemkeng F Teuwafeu D Boombhi J Menanga A. Incidence of venous thromboembolism, risk factors and prophylaxis in hospitalized patients in the south west region of Cameroon. Vasc Health Risk Manag. (2020) 16:317–24. doi: 10.2147/VHRM.S205935 32801728 PMC7383042

[4] Modi S Deisler R Gozel K Reicks P Irwin E Brunsvold M. Wells criteria for DVT is a reliable clinical tool to assess the risk of deep venous thrombosis in trauma patients. World J Emerg Surg. (2016) 11:24. doi: 10.1186/s13017-016-0078-1, PMID: 27279896 PMC4898382

[5] Stein PD Beemath A Olson RE. Trends in the incidence of pulmonary embolism and deep venous thrombosis in hospitalized patients. Am J Cardiol. (2005) 95:1525–6. doi: 10.1016/j.amjcard.2005.02.030 15950590

[6] Okuhara A Navarro TP Procópio RJ Leite JO. Incidence of deep venous thrombosis and stratification of risk groups in a university hospital vascular surgery unit. J Vasc Bras. (2015) 14:139–44.

[7] Beckman MG Hooper WC Critchley SE Ortel TL. Venous thromboembolism: a public health concern. Am J Prev Med. (2010) 38:S495–501. doi: 10.1016/j.amepre.2009.12.017 20331949

[8] Ji QY Wang MF Su CM Yang QF Feng LF Zhao LY. Clinical symptoms and related risk factors in pulmonary embolism patients and cluster analysis based on these symptoms. Sci Rep. (2017) 7:14887. 29097743 10.1038/s41598-017-14888-7 PMC5668424

[9] Yao J Han M Shi J Wang W Zhang J Zhang Y. Prognosis and factors 4 to 10 years after deep vein thrombosis: a long-term follow-up cohort study. Clin Appl Thromb Hemost. (2024) 30:10760296241266820. doi: 10.1177/10760296241266820, PMID: 39140994 PMC11375659

[10] Chan SM Brahmandam A Valcarce-Aspegren M Zhuo H Zhang Y Tonnessen BH. Sex differences in long-term outcomes of patients with deep vein thrombosis. Vascular. (2023) 31:994–1002. doi: 10.1177/17085381221097746, PMID: 35502988

[11] McLendon K Goyal A Attia M. Deep venous thrombosis risk factors. Treasure Island (FL): StatPearls Publishing (2024). 29262230

[12] Patel VB Ghosh LM Vaishnav B. Deep vein thrombosis risk stratification in intensive care unit patients: a pressing need. Int J Res Med Sci. (2020) 8:406. doi: 10.18203/2320-6012.ijrms20200217

[13] al-Ogaili A Quintero LD Adum JPS Fuentes HE Caprini J. Venous thromboembolism risk stratification: the missing link in hospitalized patients. J Atheroscler Thromb. (2018) 25:1087–8. doi: 10.5551/jat.ED096, PMID: 29731505 PMC6224207

[14] Tran HA Gibbs H Merriman E Curnow JL Young L Bennett A. New guidelines from the thrombosis and Haemostasis Society of Australia and New Zealand for the diagnosis and management of venous thromboembolism. Med J Aust. (2019) 210:227–35. doi: 10.5694/mja2.50004 30739331

[15] ten Cate-Hoek AJ Amin EE Bouman AC Meijer K Tick LW Middeldorp S. Individualised versus standard duration of elastic compression therapy for prevention of post-thrombotic syndrome (IDEAL DVT): a multicentre, randomised, single-blind, allocation-concealed, non-inferiority trial. Lancet Haematol. (2018) 5:e25–33. doi: 10.1016/S2352-3026(17)30227-2, PMID: 29217387

[16] Heit JA Spencer FA White RH. The epidemiology of venous thromboembolism. J Thromb Thrombolysis. (2016) 41:3–14. doi: 10.1007/s11239-015-1311-6, PMID: 26780736 PMC4715842

[17] Cushman M. Epidemiology and risk factors for venous thrombosis. Semin Hematol. (2007) 44:62–9. doi: 10.1053/j.seminhematol.2007.02.004, PMID: 17433897 PMC2020806

[18] Anderson FA Jr Spencer FA. Risk factors for venous thromboembolism. Circulation. (2003) 107:I9–I16. doi: 10.1161/01.CIR.0000078469.07362.E6, PMID: 12814980

[19] Zaidi SRH Rout P. Interpretation of Blood Clotting Studies and Values (PT, PTT, aPTT, INR, Anti-Factor Xa, D-Dimer) [Updated 2024 Jun 8]. In: StatPearls. Treasure Island (FL): StatPearls Publishing (2024). 38861642

[20] Alemu T Adule A Sorato MM Borsamo A. Incidence and factors associated with deep vein thrombosis among hospitalized adult patients at Hawassa university comprehensive specialized hospital Hawassa city, Sidama, Ethiopia: retrospective cohort study. J Thromb Thrombolysis. (2024) 57:164–74. doi: 10.1007/s11239-023-02889-5, PMID: 37704907

[21] Luo Z Chen W Li Y Wang X Zhang W Zhu Y. Preoperative incidence and locations of deep venous thrombosis (DVT) of lower extremity following ankle fractures. Sci Rep. (2020) 10:10266. doi: 10.1038/s41598-020-67365-z, PMID: 32581237 PMC7314767

[22] Mugeni R Nkusi E Rutaganda E Musafiri S Masaisa F Lewis KL. Proximal deep vein thrombosis among hospitalised medical and obstetric patients in Rwandan university teaching hospitals: prevalence and associated risk factors: a cross-sectional study. BMJ Open. (2019) 9:e032604. doi: 10.1136/bmjopen-2019-032604, PMID: 31772101 PMC6887052

[23] Vazquez FJ Posadas-Martinez ML Boietti B Giunta D Gandara E. Prevalence of deep vein thrombosis in hospitalized patients with suspected pulmonary embolism ruled out by multislice CT angiography. Clin Appl Thromb Hemost. (2018) 24:360–3. doi: 10.1177/1076029617696580, PMID: 28301914 PMC6714683

[24] Lawall H Oberacker R Zemmrich C Bramlage P Diehm C Schellong SM. Prevalence of deep vein thrombosis in acutely admitted ambulatory non-surgical intensive care unit patients. BMC Res Notes. (2014) 7:431. doi: 10.1186/1756-0500-7-431, PMID: 24996222 PMC4105515

[25] George AJ Nair S Karthic JC Joseph M. The incidence of deep venous thrombosis in high-risk Indian neurosurgical patients: need for early chemoprophylaxis? Indian J Crit Care Med. (2016) 20:412–6. doi: 10.4103/0972-5229.186223, PMID: 27555696 PMC4968064

[26] Wei L. Zhang X Nie Z Yu Y Xiao L. A meta-analysis: the DVT prevalence and its risk factors among SCI patients. Int J Clin Exp Med. (2017) 10:9818–27.

[27] Decousus H Quéré I Presles E Becker F Barrellier MT Chanut M. Superficial venous thrombosis and venous thromboembolism: a large, prospective epidemiologic study. Ann Intern Med. (2010) 152:218–24. doi: 10.7326/0003-4819-152-4-201002160-00006 20157136

[28] Mwandama CK Andrews B Lakhi S. Prevalence of deep vein thrombosis and associated factors in adult medical patients admitted to the university teaching hospital. Lusaka, Zambia Ann Intern Med. (2016) 43:224–30.

[29] White C Noble S Swan F Watson M Allgar V Napier E. ‘Hospice inpatient deep vein thrombosis detection (HIDDen) in advanced non-malignant diseases’: a longitudinal pilot study. BMJ. (2022) 12:e767–70. doi: 10.1136/bmjspcare-2019-002039 32046963

[30] Gearhart MM Luchette FA Proctor MC Lutomski DM Witsken C James L. The risk assessment profile score identifies trauma patients at risk for deep vein thrombosis. Surgery. (2000) 128:631–40. doi: 10.1067/msy.2000.108224 11015097

[31] Sandoval JA Sheehan MP Stonerock CE Shafique S Rescorla FJ Dalsing MC. Incidence, risk factors, and treatment patterns for deep venous thrombosis in hospitalized children: an increasing population at risk. J Vasc Surg. (2008) 47:837–43. doi: 10.1016/j.jvs.2007.11.054, PMID: 18295440

[32] Koren O Nasser A Elias M Avraham G Freidberg N Saliba W. Low venous thromboembolism incidence in high risk medical patients in an Israeli hospital. Can risk assessment be extrapolated to different populations? PLoS One. (2020) 15:e0235683. doi: 10.1371/journal.pone.0235683, PMID: 32628725 PMC7337280

[33] Nitta D Mitani H Ishimura R Moriya M Fujimoto Y Ishiwata S. Deep vein thrombosis risk stratification. Int Heart J. (2013) 54:166–70. doi: 10.1536/ihj.54.166 23774241

[34] Alikhan R Cohen AT Combe S Samama MM Desjardins L Eldor A. Prevention of venous thromboembolism in medical patients with enoxaparin: a subgroup analysis of the MEDENOX study. Blood Coagul Fibrinolysis. (2003) 14:341–6. doi: 10.1097/00001721-200306000-00004 12945875

[35] Roch AM Maatman TK Carr RA Colgate CL Ceppa EP House MG. Venous thromboembolism in necrotizing pancreatitis: an underappreciated risk. J Gastrointest Surgery. (2019) 23:2430–8. doi: 10.1007/s11605-019-04124-0, PMID: 30734182

[36] Aksu K Donmez A Keser G. Inflammation-induced thrombosis: mechanisms, disease associations and management. Curr Pharm Des. (2012) 18:1478–93. doi: 10.2174/138161212799504731, PMID: 22364132

[37] Hirsch DR Ingenito EP Goldhaber SZ. Prevalence of deep venous thrombosis among patients in medical intensive care. JAMA. (1995) 274:335–7. doi: 10.1001/jama.1995.03530040063042 7609264

[38] Huang Y Ge H Wang X Zhang X. Association between blood lipid levels and lower extremity deep venous thrombosis: a population-based cohort study. Clin Appl Thromb Hemost. (2022) 28:10760296221121282. doi: 10.1177/10760296221121282, PMID: 36189865 PMC9530559

[39] Welty FK. How do elevated triglycerides and low HDL-cholesterol affect inflammation and atherothrombosis?. Current cardiology reports(2024) 15:1–3. 10.1007/s11886-013-0400-4 PMC4465984 23881582

[40] Ugaki H Enomoto T Fujiwara K Kimura T Kawasaki T. Safety and efficacy of lower-dose unfractionated heparin for prophylaxis of deep vein thrombosis and pulmonary embolism in an Asian population. Blood Coagul Fibrinolysis. (2008) 19:585–9. doi: 10.1097/MBC.0b013e32830708ad, PMID: 18685443

[41] Mohamed AAH Othman WN El Alphy BS Sheble AM. Effect of implementing nursing care guidelines on the occurrence of deep vein thrombosis among orthopedic patients. IOSR J Nurs Health Sci. (2017) 6:28–36. doi: 10.9790/1959-0603012836

